# Effects of different extracts of curcumin on TPC1 papillary thyroid cancer cell line

**DOI:** 10.1186/s12906-018-2125-9

**Published:** 2018-02-15

**Authors:** Angelica Perna, Antonio De Luca, Laura Adelfi, Tammaro Pasquale, Bruno Varriale, Teresa Esposito

**Affiliations:** 1 Department of Mental and Physical Health and Preventive Medicine, Section of Human Anatomy, University of Campania “Luigi Vanvitelli”, Via Costantinopoli 16, 80138 Naples, Italy; 2 Department of Experimental Medicine, Section of Human Physiology, and Unit of Dietetic and Sport Medicine, University of Campania “Luigi Vanvitelli”, Via Costantinopoli 16, 80138 Naples, Italy; 3 Department of Experimental Medicine, Molecular Genetics Laboratory, University of Campania “Luigi Vanvitelli”, Via Costantinopoli 16, 80138 Naples, Italy

**Keywords:** Curcumin, Thyroid, TPC-1 cells, Anti-oxidant, Nutraceutical

## Abstract

**Background:**

The thyroid gland is one of the largest endocrine glands in the body. The vast majority of TCs (> 90%) originate from follicular cells and are defined as differentiated thyroid cancers (DTC) and the two histological subtypes are the papillary TC with its variants and the follicular TC. Curcumin possesses a wide variety of biological functions, and thanks to its properties, it has gained considerable attention due to its profound medicinal values (Prasad, Gupta, Tyagi, and Aggarwal, Biotechnol Adv 32:1053–1064, 2014). We have undertaken the present work in order to define the possible role of curcumin in modulating the genetic expression of cell markers and to understand the effectiveness of this nutraceutical in modulating the regression of cancer phenotype.

**Methods:**

As a template we used the TPC-1 cells treated with the different extracts of turmeric, and examined the levels of expression of different markers (proliferative, inflammatory, antioxidant, apoptotic).

**Results:**

Treatment with the three different curcumin extracts displays anti-inflammatory, antioxidant properties and it is able to influence cell cycle with slightly different effects upon the extracts. Furthermore curcumin is able to influence cell metabolic activity vitality.

**Conclusions:**

In conclusion curcumin has the potential to be developed as a safe therapeutic but further studies are needed to verify its antitumor ability in vivo.

## Background

The thyroid gland is one of the largest endocrine glands in the body. It controls metabolism, protein synthesis, and body’s sensitivity to other hormones. It participates in these processes by producing thyroid hormones, the principal ones being thyroxine (T_4_) and triiodothyronine (T_3_), which is the active hormone. These hormones regulate the growth and rate of function of many other systems in the body, mediated by thyroid hormone receptors [1]. Hormonal crosstalk also plays a key role in regulating thyroid functioning [2, 3]. Thyroid cancer (TC) is the most common endocrine malignancy and the fifth most common cancer diagnosed in women. It has been reported that its incidence has the largest annual increase in men and women amongst all cancers in the United States. The vast majority of TCs (> 90%) originate from follicular cells and are defined as differentiated thyroid cancers (DTC) and the two histological subtypes are the papillary TC with its variants and the follicular TC. Differentiated thyroid cancer is usually an indolent disease that with adequate treatment has an excellent prognosis [4].

Papillary thyroid carcinoma is often non-enveloped and multifocal, i.e., it simultaneously affects different parts of the thyroid gland, and spreads mainly because of lymph nodes. It is characterized by the presence of buds, psammoma body, inclusion bodies and enlarged nuclei with irregular contour and a central notch. Papillary thyroid carcinoma accounts for about 80% of thyroid cancers and gives lymph node metastases with limited location, often for a long time, only to neck lymph nodes, only after the cancer metastasizes outside the neck. For this reason, the papillary carcinoma may have lymph node metastases in 30% of cases, already at the time of diagnosis [5, 6].

The condition of obesity, both in adults and children, and the association of a malfunction on the part of orexin, can accentuate the possibility of thyroid inflammation [7, 8]. The lipid metabolism which plays an important role on the thyroid metabolism, is regulated by the amount and quality of unsaturation of the lipids [8].

Curcumin [diferuloylmethane: (1E, 6E) -1,7-bis (4-hydroxy-methoxyphenyl) -1,6-heptadiene-3, 5-dione] is the active ingredient of the dietary spice found in the rhizomes of *Curcuma longa (Curcuma longa L.)*, a plant in the ginger family. Turmeric, a common oriental spice that gives curry powder its yellowish color, is frequently used in Asian cooking, particularly Indian, Pakistani, and Thai cooking. The active ingredients are made up mainly by curcuminoids, demethoxycurcumin, bisdemethoxycurcumin and curcumin. In particular curcumin is a lipophilic phenolic substance that has attracted great attention from the scientific community for its many beneficial biological properties [9]. For its intense yellow color curcuma is used for dying purposes, especially in the food industry. Numerous studies have indicated that curcumin possesses a wide variety of biological functions, such as anti-inflammatory, anti-cancer, anti-oxidant, antimicrobial, wound-healing and hypoglycemic activities [10]. A series of epidemiological studies have shown a potential activity of curcumin in several diseases [11]; it is able to modulate expression levels of proteins involved in many cellular processes and then to regulate transcription, apoptosis, proliferation. This is the reason why it has been subject to several studies aimed at changing in its administration form that have permitted an increase in bioavailability and effectiveness against different diseases, decreasing the mortality and morbidity associated to these pathologies. There are several plant extracts of Turmeric which may be employed in therapeutic practice: Arjuna®, Naturex®, C3Complex® DC 95% and so on.

As far as it concerns the papillary thyroid cancer, TPC1 cell is the most common cell line used for investigation. This cell line is well defined for its genetic properties similar to the in vivo papillary thyroid cancer [12]. In this respect we have undertaken the present work in order to define the possible role of curcumin in modulating the genetic expression of cell markers and to understand the effectiveness of this nutraceutical in modulating the regression of cancer phenotype.

## Methods/design

### Cell line and culture conditions

Papillary thyroid cancer cell line TPC1 was a kindly gift of Prof. Santoro from the University of Naples “Federico II”. TPC1 is a validated model of human papillary thyroid cancer (PTC), in which both RET/PTC1 rearrangement and BRAF V600E mutation [13], the most common genetic alterations detected in human PTC [14], are present. The cells were cultured and maintained in RPMI 1640 culture medium (Gibco) containing 10% FBS (foetal bovine serum, Gibco), 100 U/ml penicillin and 100 U/ml streptomycin (Gibco) in a humid atmosphere of 5% (*v*/v) CO_2_ and 95% (v/v) air at 37 °C. Cells were treated with the three different extracts of curcumin (Arjuna® Natural Extracts Ltd. BCM-95, Naturex® Ultimate Botanical Benefits, Curcumin C3Complex® DC 95% Lot.No 14/01711) (Table 1) at a concentration of 25 μM, dissolved in the culture medium, for 24-48 h as previously described [15]. Control cells were treated with the same medium and solvent but without the three different extracts of curcumin. The solvent control contained an equivalent amount of DMSO (dimethyl sulfoxide, Sigma) corresponding to the highest concentration of curcumin used. Cells were detached with trypsin/EDTA (Gibco) and then replated in cell culture plates. Each of the three different extracts was dissolved in DMSO at 5 × 10^−3^M, and stored at 20 °C until dilution before use. All turmeric extracts were provided by Biosalus Company (Naples, Italy).

**Table 1:** Products Specification

Arjuna®	Extraction: Ethyl Acetate 100%	Total Curcuminoids: 86% Curcumin: 65% Composition: Curcumin, Demethoxy Curcumin, Bis-Demethoxy Curcumin, Essential Oils of turmeric rhizome (Ar-curcumene, α-curcumene, Zingeberene, β-sesuiphellandrine, β-atlantone, Germacrone)
Naturex®	Extraction: Acetone 100%	Total Curcuminoids: 85–97% Curcumin: 70–80% Composition: Curcuminoids, Curcumin, Demethoxy Curcumin, Bis-Demethoxy Curcumin
C3Complex®	Extraction: Ethyl Acetate, Iosopropyl Alcohol	Total Curcuminoids: 95,73% Curcumin: 80,87% Composition: Curcumin, Demethoxy Curcumin, Bisdemethoxy Curcumin

### Cell viability assay

The effects of the three different extracts of curcumin on cell viability were determined by the MTT assay as previously described with some modifications [16]. In brief, cells (10,000 cells per well) were incubated with or without the three different extracts of curcumin in three different experiments in a 96-well plate and incubated for 24-48 h at 37 °C. Our control is given by cycling TPC1 cells. After incubation, 10 μl of MTT (3 [4,5 dimethylthiazol 2yl] 2,5 di-phenyl-tetrazolium bromide, GoldBio.Com) solution (5 mg/ml) were added to each well and incubated for another 4 h at 37 °C. The supernatants were aspirated carefully and 100 μl of DMSO were added; then the plate was held on vibrator for 20s. The optical density of the cell suspension was measured at 570 nm using a microplate reader (Bio-Tek instruments Inc., Winooski, VT). Cell viability was expressed as a percentage of MTT reduction, assuming that the absorbance of untreated cells was 100%.

## Western blot assay

TPC1 cells pretreated with different types of extract of curcumin at 25 μM were incubated for 24-48 h, and then western blot analyses were carried out as previously described with some modifications [17]. A sufficient number of cells have been plated so as to obtain a protein concentration sufficient to carry out the technique by loading 20 μg of protein per line [18]. Our control is given by cycling cells. After centrifugation, cells were lysed in 50 μl of lysis buffer (Ripa Lysis buffer, Santa Cruz Technologies, Inc., Santa Cruz, CA) containing protease inhibitor (Protease Inhibitor Cocktail-Santa Cruz Technologies, Inc., Santa Cruz, CA), PMSF (Santa Cruz Technologies, Inc., Santa Cruz, CA) and Sodium Orthovanadate, (Santa Cruz Technologies, Inc., Santa Cruz, CA) supplement for 15 min in ice. The lysate was centrifuged at 12,000 g for 5 min at 4 °C. The supernatant was collected and the protein concentration was determined using Protein Assay (Bio-Rad Laboratories, Segrate Milan, Italy). After addition of sample loading buffer, protein samples were analyzed with electrophoresis on 10% SDS–PAGE and subsequently transferred onto a nitrocellulose membrane (Millipore Corp, Bedford, MA, USA). The membrane was incubated in fresh blocking buffer [0.1% (*v*/v) Tween 20 in Tris-buffered saline, pH 7.4, containing 5% (*w*/*v*) skim milk] at room temperature for 1 h and then probed with the following antibodies: anti-p53 (1:500, *v*/v) (Calbiochem, LaJolla, CA, USA), anti-β-catenin (1:500, v/v) (BD-Biosciences Phamingen, United States); instead from Santa Cruz biotechnology, California, USA we purchased: p21 (1:200, *v*/v), anti-cyclinD1 (1:500, v/v), anti-pro-caspase3 (1:500, v/v), anti-VEGF (1:500, v/v), anti-Bcl2 (1:500, v/v), anti-TNFα (1:500, v/v), anti-Nrf2 (1:500, v/v) in blocking buffer at 4 °C overnight. After three times washing, each for 5 min with TBS-T (Tris-buffered saline with 0.1% (v/v) Tween 20) (Sigma Chemical Company, St. Louis, MO), the membrane was incubated with the appropriate HRP-conjugated secondary antibody (Goat anti-mouse IgG, 1:5000 or Goat anti-rabbit IgG, 1:5000 (Immunoreagents, Inc.) or Donkey anti-goat IgG, 1:5000 (Promega, Madison, WI, U.S.A.) at room temperature for 1 h and then washed again three times in TBS-T buffer. The membrane was incubated with enhanced chemiluminescence substrate solution: Super Signal West Pico (Thermo Scientific, Italy) for 5 min according to the manufacturer instructions and visualized with autoradiography film. Each membrane was probed with the monoclonal anti-β-tubulin antibody (1:500, *v*/v) (Sigma Chemical Company, St. Louis, MO), to estimate equal protein loading. Quantitation of membranes was made using ImageJ software. The quantitation of bands was made using the relationship between the intensity of the bands with the intensity of β-tubulin.

### Statistical analyses

Where applicable, results were expressed as the mean ± SEM of at least three independent experiments. Statistical differences were considered if the *p* value was < 0.05 *, *p* value was < 0.01**, *p* value was < 0.001*** as determined by ANOVA followed by Student’s t-test. Data are expressed as the percentage and SEM.

## Results

Curcumin displays both anti-inflammatory and antioxidant properties, thus having a potential role in the development of cancer preventive strategies and applications in clinical research. It derives from the rhizome *Curcuma longa L.* and can be obtained with different methods of extraction. In the present study we have evaluated the effects of three different extracts of Curcuma, Arjuna®, Naturex® and C3Complex® on TPC1 cells.

**Fig. 1:**
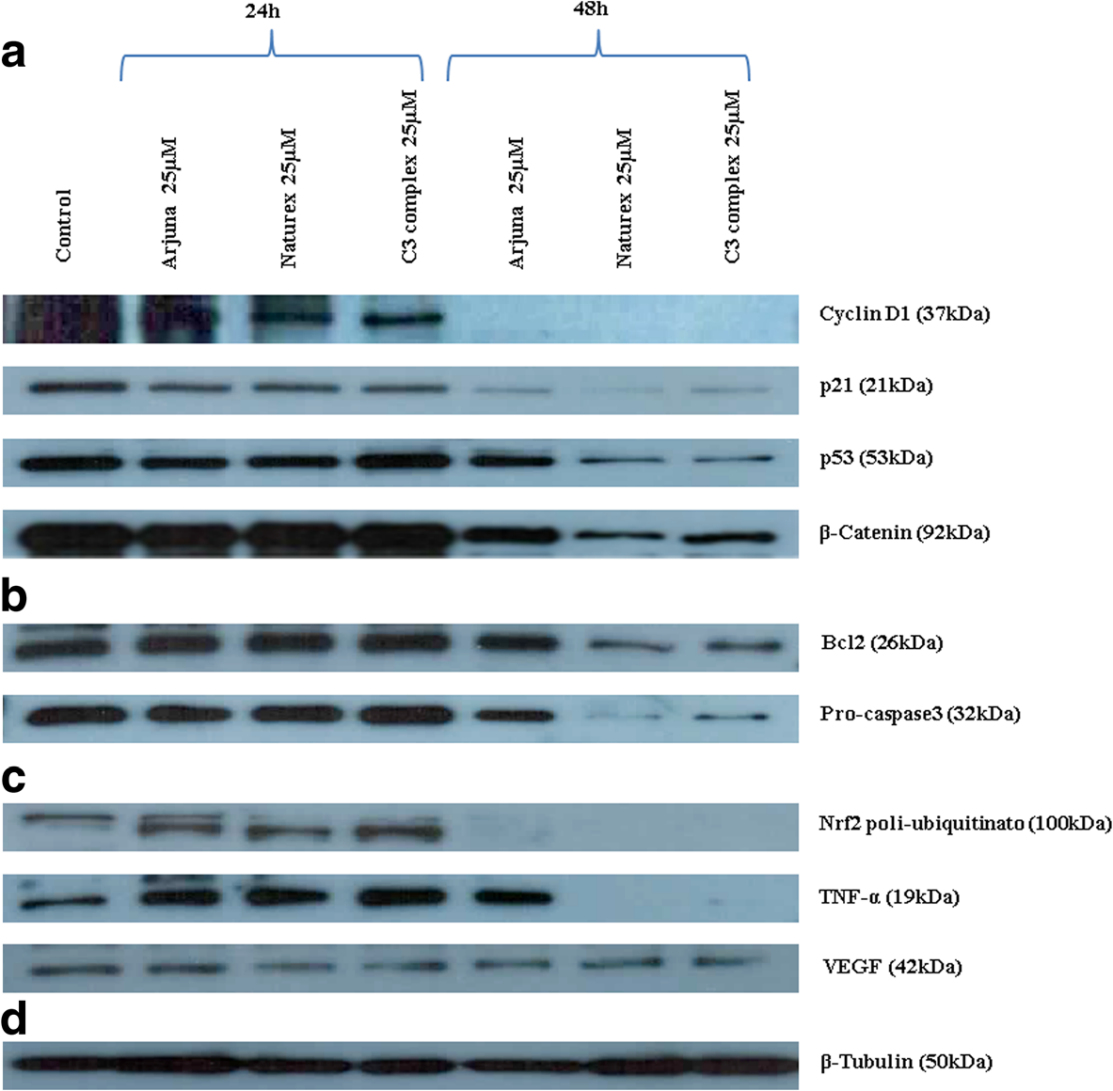
Effects of three different extracts of Curcuma (Arjuna®, Naturex®, C3Complex®) on a papillary thyroid carcinoma cell line (TPC1), treated with 25 μM for 24-48 h. Data obtained by Western blot, analyzing Cyclin D1, p21, p53, β-Catenin (**a**), Bcl2, pro-Caspase3 (**b**), Nrf2, Tnf-α, VEGF (**c**), β-tubulin (**d**)

**Fig. 2:**
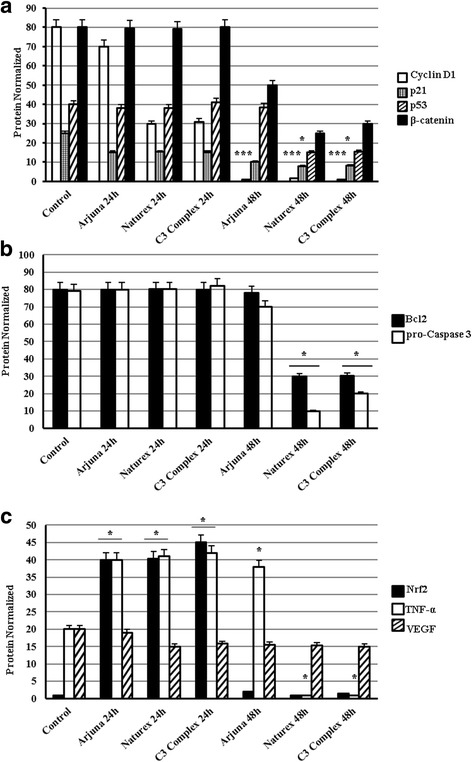
Western blot analysis of the expression level of Cyclin D1, p21, p53, β-Catenin (**a**), Bcl2, pro-Caspase3 (**b**), Nrf2, Tnf-α, VEGF (**c**) of cells pretreated with the three different extracts of Curcuma (Arjuna®, Naturex®, C3Complex®) with 25 μM for 24-48 h. Densitometric analysis of the western-blot bands was made using ImageJ software, and from the analysis it was obtained the histogram. β-Tubulin was monitored as a loading control. All data represent as the means ± SEM of three independent experiments. *p* < 0.05*, *p* < 0.01**, *p* < 0.001*** vs. the control (untreated cells) (ANOVA)

In order to understand the effects of these extracts on the thyroid cell line we treated the cells with the three extracts for 24 and 48 h at a final concentration of 25 μM. Our control is given by cycling TPC1 cells. After treatment we examined their properties as modulators of the cell cycle, as antioxidant and anti-inflammatory agents. As showed in Fig. 1a proteins involved in cell cycle progression were modulated after treatment. In particular we observed a non-significant decrease of cyclin D1 at 24 h compared with the untreated cells, instead at 48 h the expression of cyclin D1 is completely inhibited compared with the untreated cells (*p* < 0.001) (Figs. 1a and 2a). In cells treated with the three different extracts of curcumin we observed a down regulation of p21 and p53, whose accumulation was low at 24 h and higher at 48 h, significant for p21 in cells treated with Naturex® and C3Complex® (*p* < 0.05) (Figs. 1a and 2a). Interestingly they were able to inhibit at 48 h β-catenin, a membrane-bound cell growth-signaling complex that plays a role in cell adhesion, as well as in the promotion of growth (Figs. 1a and 2a). A comparison among the three extracts highlighted that Naturex®, even though there are no great differences and all the extracts are similar, was always the most effective.

Furthermore we examined if the extracts of curcumin were able to modulate key proteins of the apoptosis. Always by immunoblotting we observed that accumulation of Bcl2, which is a survival gene, was not different compared with the untreated cells at 24 h. At 48 h, Arjuna® still does not have any effect on the expression of Bcl2, instead Naturex® and C3Complex® are able to inhibit the expression (*p* < 0.05) (Figs. 1b and 2b). The Naturex® and C3Complex® ability to inhibit the accumulation of Bcl2 was consistent with the ability of these two extracts to activate the pro-caspase3. These effects on the thyroid cells were independent of p53 and of the block of the cell cycle.

As showed in Figs. 1c and 2c we evaluated the antioxidant and inflammatory effects of these extracts using two major markers, Nrf2, which regulate the expression of antioxidant proteins protecting against oxidative damage triggered by injury and inflammation, and TNFα, involved in acute inflammations. It is seen that in cancer cells there are high levels of Nrf2 expression and this expression seems regulated by p21 [19]. By immunoblotting assay we observed at 24 h the polyubiquitated form of Nrf2 (*p* < 0.05), while at 48 h it is completely degraded. This seems to influence the expression of VEGF [20] which shows a low down regulation, both at 24 h and 48 h.

Immunoblotting assay showed that the three extracts were able to inhibit TNFα with different effects between the three extracts. Specifically we observed that expression of TNFα at 24 h was increased compared with the untreated cells (*p* < 0.05), instead at 48 h the expression of TNFα was still higher in the cells treated with Arjuna® (*p* < 0.05), while it was completely absent in the cells treated with Naturex® and C3Complex® (*p* < 0.05).

All these experiments point out that treatment with the three different curcumin extracts displays anti-inflammatory, antioxidant properties and it is able to influence cell cycle with slightly different effects upon the three extracts examined.

**Fig. 3:**
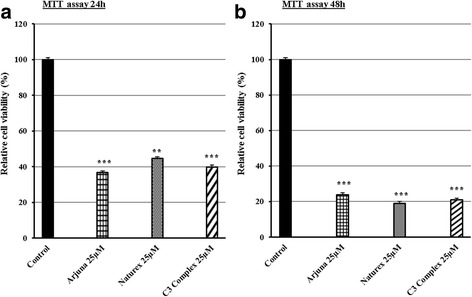
Effects of three different extracts of Curcuma, Arjuna®, Naturex®, C3Complex® on a papillary thyroid carcinoma cell line (TPC1), treated with 25 μM for 24 h (**a**) and 48 h (**b**). After incubation, the effects of curcumin on cell viability were determined by MTT assay. The optical density of the cell suspension was measured at 570 nm using a microplate reader, and from the analysis it was obtained the histogram. All data represent as the means ± SEM of three independent experiments. *P* < 0.01**, *P* < 0.001*** vs. the control (untreated cells) (ANOVA)

Altogether these data have highlighted also that curcumin is able to influence cell metabolic activity vitality. Indeed to evaluate the vitality after treatment we measured the survival of TCP1 cells by a MTT assay. Figure 3a showed a reduction of viability, for all three extracts, more of 50% compared with the untreated cells within the first 24 h. In particular we observed that the reduction of viability was more evident in the Arjuna® and C3Complex® (*p* < 0.001) compared with Naturex® (*p* < 0.01). The effects were observed also at 48 h for all three extracts; a long treatment demonstrated that Arjuna® and C3Complex® were almost able to reach the effects induced by Naturex® (*p* < 0.001) (Fig. 3b).

## Discussion

Because of the properties of curcumin on cancer cells, we investigated its effects in TPC1 cells, a cellular model of papillary thyroid cancer [12]. Three different extracts were used and it was assessed whether the effects were comparable. Arjuna® is the product of a unique extraction process that brings the curcuminoids and essential oils, like curcumin and Ar-tumerone, to the forefront. The composition of this potent curcumin-essential oil complex (CEC) mixture is given by 86% curcuminoids, plus up to 7% essential oils. C3Complex®, collectively known as curcuminoids, is a standardized preparation (Sabinsa) of three curcuminoids; Curcumin (76.07%), Demethoxycurcumin (DMC; 20.28%), and Bis-Demethoxy Curcumin (3.63%). The safety and efficacy of C3Complex® is established in clinical trials [21–23]. Naturex® is a preparation of three curcuminoids extracted with natural processes; Curcumin (70–80%), Demethoxy Curcumin (15–25%), Bis-Demethoxy Curcumin (2.5–6.5%). Our data show for the first time that curcumin-enriched compounds are able to decrease TPC1 cell survival and this occurs through the induction of apoptosis mainly by significantly reducing the accumulation of Bcl2 and cyclin D1 and the levels of p21 and p53. Besides, β-catenin, involved in cell growing, is also reduced by these curcumin-enriched compounds. It is also noteworthy that Nrf2, a downstream target of p21, is also affected.

Several studies established that curcumin modulates various molecular targets including transcription factors, growth factors and their receptors, cytokines, enzymes, and genes regulating, cell proliferation and apoptosis [10]. Thanks to all these activities, curcumin is positioned as an interesting nutraceutical.

Indeed curcumin has been shown to be a potent agent that has interesting effects in various central nervous system (CNS) disorders including Alzheimer, Parkinson, and stroke [24]. Moreover a recent study has shown that curcumin is able to cross the blood brain barrier and inhibit tumor growth in orthotopic glioblastoma models [25]. Actually curcumin has potential antitumor effects in a large variety of cancers [26].

After treatment we examined the three extracts’ properties as modulators of the cell cycle, antioxidant and anti-inflammatory. In particular our screening recognized that several proteins involved in cell cycle regulation, cyclin D1 and p21, were modulated after treatment compared with the untreated cells, together with β-catenin, a membrane-bound cell growth-signaling complex that plays a role in cell adhesion and gene transcription as an intracellular signal transducer in the Wnt signaling pathway. These data point out that curcumin is able to inhibit the cell cycle progression through the cyclin D1 protein inhibition. Cyclin D1 is an oncoprotein that plays a key role in the development of all cancer cells and leads to increased cell proliferation, which gives neoplastic cells a growth advantage and may also favor the occurrence of additional genetic lesions with potential oncogenic effects.

Simultaneously curcumin attenuates the β-catenin signaling in TPC1 cells probably by promoting phosphorylation-dependent degradation of β-catenin. Curcumin is known to be a good inhibitor of the Wnt/β-catenin signaling pathway in gastric, colon, intestinal and prostatic cancer cell lines [27]. However the detailed molecular mechanisms of curcumin-mediated reduction of β-catenin are not fully understood.

It is notewhorty to mention that the Wnt/β-catenin signaling pathway plays an important role in the proliferation and differentiation of progenitor cells during brain development. Canonical Wnt signaling pathway activates GSK3ßphosphorylates and translocates nuclear ß-catenin from the nucleus to the cytoplasm resulting in the inhibition of the subsequent activation of T cell factor 4 (TCF4)-dependent gene transcription (such as cyclin D1 and c-Myc) [28].

In conclusion, these findings provide evidence that the inhibitory effect of curcumin on cell proliferation involves the inhibition of the β-catenin pathway, in concert with the down-regulation of cyclin D1, which is tightly connected to the development of TPC1 cells.

Furthermore, in our experimental model, results showed that some apoptotic markers in TPC1 cells, like Bcl2 and pro-caspase3, clearly indicate that the inhibitory effects of high levels of both Bcl2 and pro-caspase3 are abrogated by the presence of curcumin in the culture medium. This makes us hypothesize that in TPC1 cells curcumin is able to remove the “genetically settled” impairment of apoptosis mechanism. This is also confirmed by the MTT experiments, where a decrease of the cell viability is compromised by the presence in the culture medium of curcumin suggesting its probable negative effect onto progression of cell cycle. We observed an up-regulation of TNF-α at 24 h. TNF-α is one of the most significant mediators involved in tumor cell death by the induction of multiple intracellular pathways, including the generation of reactive oxygen intermediates in the mitochondria preceding plasma membrane permeabilization and the induction of iNOS expression. Ultimately, these processes may lead to cell death. This mechanism may be triggered in the first 24 h but it is not clear why at 48 h there is a complete disappearance in the cells treated with Naturex® and C3Complex®.

Curcumin is able to inhibit p53 expression. The p53 protein, also known colloquially as the “guardian of genome”, helps to eliminate DNA damage from cells following genotoxic stress by accelerating DNA repair processes and activating transient cell cycle checkpoints to facilitate repair. When the damage is severe, p53 can trigger apoptotic cell death either directly through its poly-proline region, or indirectly through transcriptionally upregulating pro-apoptotic proteins such as the BH3-only family (PUMA, NOXA and BAX), and down-regulating anti-apoptotic proteins such as BCL2 and surviving [29, 30]. Mounting evidence showed that chromosome maintenance1 (CRM1) plays an oncogenic role in human cancers [31]. CRM1 controls Nrf2 nuclear export [32] and its transcription is deregulated by wild-type p53 due to decreased NFY and Sp1 binding to CRM1 promoter [33]. Down-regulated p53 may lead to an increase of CRM1 expression, able to translocate Nrf2 from the nucleus, where it transcribes for different antioxidant factors, to the cytoplasm, where it is poly-ubiquitinated and then degraded. We observed the poly-ubiquitous form of Nrf2 at 24 hby immunoblotting assay, while at 48 h it is completely degraded. This seems to influence the expression of VEGF [20] which shows a low down-regulation, both at 24 h and 48 h. Several studies have shown a deleterious aspect of Nrf2 [34]. Its high and prolonged activation in cancer cells has been long associated with progression, metastatic invasion, angiogenesis, and chemo and radio-resistance in tumors and is considered a poor prognostic factor [35]. Indeed, the stable over-expression of Nrf2 was found in various types of tumors such as lung [36], breast [37], head and neck [38], ovarian [39] and endometrial cancer [40]. Evidence shows that different proteins can alter the Nrf2-Keap1 binding [41]. Nrf2 activity is subject to a positive regulation by p21 [19], which interferes with Keap1-mediated ubiquitination, interacting with the DLG motif in Nrf2, leading to its stabilization. Therefore, Nrf2 expression is significantly lower in the absence of p21, and conversely it is increased upon p21 over-expression. In this respect, our results fit well with the above cited facts. The expression of Nrf2 results completely abrogated in TPC1 cells after the treatment with curcumin in 48 h, with the consequent down-regulation of p21 protein.

Among the expression of the pro-angiogenic VEGF protein, our results in TPC1 cells show that the presence of curcumin in the culture medium results in a slight reduction of VEGF expression. These data according to the fact that novel synthetic curcumin analogs have shown to be potent antiangiogenic agents in colorectal cancer [42], indicated that in the TPC1 cells have a similar effect. Thus, there may be other mechanisms involved in the anti-proliferative effect of curcumin.

## Conclusions

In conclusion, these findings provide evidence that the inhibitory effect of curcumin on cell proliferation involves the inhibition of the β-catenin pathway reducing nuclear β-catenin, accompanied by the down-regulation of cyclin D1, which is tightly connected to the proliferation of TPC1 cells.

Curcumin, a key component in functional foods, has been used for centuries, and was reported to promote health and fight against several human diseases including cancers. Numerous studies indicated that curcumin may inhibit the growth of many cancer types. However, the predominant pathway involved in curcumin induced tumor suppression appears to be highly cell type and context dependent. Curcumin can trigger p53-dependent [43] and p53-independent apoptosis in tumor cells [44], thus, the molecular mechanisms of the anticancer activities of curcumin are numerous and varied, depending on the cancer cell type or cell environment.

For these reasons we can assert that curcumin has the potential to be developed as a safe therapeutic but further studies are needed to verify its antitumor ability in vivo.

Considering the three different curcuma extracts, and comparing the different activities of these, the difference is supposed to come from the various components present in them. While Naturex® and C3Complex® extracts are very similar to each other, since the only components present are curcuminoids (Curcumin, Demethoxy Curcumin e Bis-Demethoxy Curcumin), in the Arjuna® extract, which has the lowest activity, there are also essential oils (Ar-curcumene, α-curcumene, Zingeberene, β-sesuiphellandrine, β-atlantone, Germacrone). No interactions that may result from the presence of such components are known. As it is known that the combination of curcumin with other nutraceuticals can increase bioavailability and hence the activity [10], the opposite could be plausible, i.e. the possibility that interacting with other components reduces the activity.

## Abbreviations

CNS: Central nervous system
CRM1: Chromosome maintenance1
DMSO: Dimethyl sulfoxide
MTT: 3 [4,5 dimethylthiazol 2yl] 2,5 di-phenyl-tetrazolium bromide
PTC: Human papillary thyroid cancer
TBS-T: Tris-buffered saline with 0.1% (*v*/v) Tween 20
TCF4: T cell factor 4
TPC1: Papillary thyroid cancer cell line
